# *Bsep* expression in hilar cholangiocarcinoma of rat model

**DOI:** 10.1038/s41598-021-82636-z

**Published:** 2021-02-03

**Authors:** Meng-yu Zhang, Jie-ping Wang, Kai He, Xian-ming Xia

**Affiliations:** 1 Department of Hepatobiliary Surgery, The Affiliated Hospital of Southwest Medical University, Luzhou, 646000 Sichuan Province China; 2 Department of Rehabilitation, The Affiliated Hospital of Southwest Medical University, Luzhou, 646000 China

**Keywords:** Cancer models, Cancer

## Abstract

Develop a rat model of hilar cholangiocarcinoma for detecting bile salt export pump (*Bsep*) expression in hilar cholangiocarcinoma tissues, in order to provide a new therapeutic target for the gene therapy of hilar cholangiocarcinoma. Sixty male Wistar rats (body weight, 190 ± 8 g) were randomly divided into three groups (the experimental group, the control group and the sham operation group, n = 20 each) as follows: The three groups were fed a standard diet, the experimental group was injected by cholangiocarcinoma QBC939 cell suspension along the hilar bile duct into the bile duct bifurcation with microsyringe, the control group was injected by normal saline, the sham operation group did not inject anything. Every day assess the rats’ mental state, diet, and motion by using Basso–Beattie–Bresnahan and combined behavioral score. At 4 weeks, one rat of the experimental group was sacrificed after it was administered anesthesia, and we recorded changes in hilar bile duct size, texture, and form. This procedure was repeated at 6 weeks. After 6 weeks, hilar cholangiocarcinoma developed only in the experimental group, thereby establishing an experimental model for studying QBC939-induced hilar cholangiocarcinoma. Tumor formation was confirmed by pathological examination, and hilar bile duct tissues were harvested from both the groups. A real-time polymerase chain reaction assay and an immunohistochemical assay were used to analyze the expression of *Bsep* in hilar bile duct tissues of each group. From the second week, the rats in experimental group began to eat less, and their body mass decreased compared with control group and sham operation group. After 6 weeks, we detected hilar cholangiocarcinoma in the hilar bile duct tissues of 18 rats (90%) in the experimental group. In the experimental group with hilar cholangiocarcinoma, we found that the levels of total cholesterol, total bilirubin, and direct bilirubin were higher compared with those in the control group and sham operation group. Simultaneously, muddy stones emerged from the bile ducts of rats in the experimental group. The *Bsep/Gapdh* mRNA ratio in hilar cholangiocarcinoma, control group and sham operation group differed markedly. Light microscopy revealed a granular pattern of *Bsep* protein expression which reacted with the anti-*Bsep* antibody. Each section was randomly divided into six regions, with 80 cells were observed in every region. Sections with > 10% positive cells were designated positive, Sections with < 10% positive cells were designated negative. Each group included 4800 cells. In the experimental group, 1200 cells (25%) were positive, in the control group, 3648 cells (76%) were positive and in the sham operation group 3598 cells (75%) were positive, and this difference was statistically significant. *Bsep* expression significantly decreased in hilar cholangiocarcinoma of rats than those in control group and sham operation group, suggesting that drugs targeting *Bsep* are a new strategy for hilar cholangiocarcinoma.

## Introduction

Hilar cholangiocarcinoma is a malignant tumor in the digestive system, and the patients who die from this disease increases every year. Unfortunately, treatment methods are limited^1–3^. The preferred method is Surgery, but it has contraindications. Only part of patients are eligible for radical resection, and some patients must undergo palliative resection, although the 5-year survival rate is low. Numerous patients are not eligible for surgery because of factors such as tumor infiltration, or metastasis, multiple organ dysfunction. Alternative therapies include radiotherapy, chemotherapy, and radiofrequency ablation; however, they do not improve survival rates. Therefore, new therapies are urgently required.

Gene therapy for cancer is not widely used, but it has great potential. Therefore, we searched for genes involved in the pathogenesis of hilar cholangiocarcinoma that may serve as therapeutic targets. Farnesyl X receptor (FXR) is related with bile acid metabolism, bile salt export pump (Bsep) is target gene of FXR^4–6^, which is important in bile acid secretion. Insook et al. found that mice without FXR gene developed cirrhosis, spontaneously formed liver cancer and even cholangiocarcinoma on the basis of cholestasis. In former experiment we have detected that FXR expression decreased in hilar cholangiocarcinoma tissues of rats, which led us to investigate the role of *Bsep*, whether the role of *Bsep* is enhanced or diminished in hilar cholangiocarcinoma. Expecting to find a new target for the treatment of hilar cholangiocarcinoma.

## Materials and methods

Statement: The study protocol was approved by the Ethics Committee of the affiliated hospital, Southwest Medical University, Luzhou, Sichuan Province, China. Animal experiments have passed ethical review and have been approved by central laboratory of the Affiliated Hospital of Southwest Medical University. Number: 2020415. All methods were carried out in accordance with animal experiment guidelines and regulations. Animal welfare guidelines abided by China Laboratory Animal Welfare Law and Animal management regulations, Number: GB/T 35892‐20181.

### Rats

It were provided by the Animal Test Center of Southwest Medical University. We randomly divided 60 Wistar rats (male, 190 ± 8 g) into three equal groups (the experimental group, the control group and the sham operation group, n = 20 each) as follows: The experimental group was injected by cholangiocarcinoma QBC939 cell suspension along the hilar bile duct into the bile duct bifurcation with microsyringe, the control group was injected by normal saline, the sham operation group did not inject anything. Before conducting the study, the rats were healthy and were not administered a diet containing drugs.

### Experimental methods

Materials: DMEM culture medium (Sigma Inc.), Pentobarbital sodium (Shanghai Xinyu Biotechnology Co., Ltd.), QBC939 human cholangiocarcinoma cell line (Shanghai Yubo Biotechnology Co., Ltd.), Microsyringe (Shanghai Fuguang Precision instrument Co., Ltd.). Frozen tissue sections were prepared using a cryostat, HFsafe biological safety cabinet, microscopic imaging system (PM-10A). The main reagents were real-time PCR kits, anti- *Bsep* monoclonal antibody. The sense and antisense primers used to detect *Bsep* mRNA were as follows: 5′-CCCTCAACTGATGGGGGCTCCAGT-3′ and 5′-CCCATGTCTGA CTCAGTGATTCTT-3′. The sense and antisense primers used to detect *Gapdh* mRNA were as follows: 5′-GATGGTGGGTATGGG TCAGAA-3′ and 5′-CTAGGAGCCAGGGCAGTAATC-3′. The 2-∆∆Ct method was used to express the data.

The tumor cells were cultured in DMEM medium at 37℃and 5% saturated humidity. Selected cells with good growth to inoculate Wistar rats. The diameter of the needle tip of the microsyringe is 40 μm, connected to the 1 ml syringe through a rubber tube.

Establish animal model The cultured cholangiocarcinoma QBC939 cells were prepared to cell suspension with a concentration of 1 × 10^6^ cells/ml. The three groups were fed a standard diet throughout the course of the study. Wistar rats in the experiment group were anesthetized with 1.5% pentobarbital sodium and 0.2 ml/100 g intraabdominal injection. After disinfection, the abdomen was cut along the linea alba. After the tumor cell suspension was adsorbed by microsyringe, the needle of microsyringe punctured along the hilar bile duct into the bile duct bifurcation, and 100 μl of tumor cell suspension was injected. Pressed to stop the bleeding, closed the abdomen in turn, and end the operation. The control group was injected by normal saline, the sham operation group did not inject anything. Every day assess the rats’ mental state, diet, and coat condition. At 4 weeks, one rat of the experimental group was sacrificed after it was administered anesthesia, and we recorded changes in hilar bile duct size, texture, and form. This procedure was repeated at 6 weeks. Tumor formation was confirmed according to the findings of pathological examination, and hilar bile duct tissues were harvested from the three groups for the analysis of *Bsep* mRNA expression using real-time PCR (RNA was extracted from cancer and normal hilar bile duct tissues using Trizol reagent. An ABI7500 real-time PCR detection system was used, and Gapdh served as the internal control. Real-time PCR kits was used (SR1100)) and *Bsep* protein expression using immunohistochemistry (anti-*Bsep* antibody was used (CHEMICON)). Light microscopy revealed a granular pattern of *Bsep* protein expression which reacted with the anti-*Bsep* antibody. Each section was randomly divided into six regions, with 80 cells were observed in every region. Sections with > 10% positive cells were designated positive, Sections with < 10% positive cells were designated negative.

### Statistical analysis

SPSS22.0 statistical software was used for data analysis. Data are presented as the mean ± SD. The *t* test was used to judge the differences between two groups, and *P* < 0.05 indicates statistically significance differences. The χ^2^ test was used to evaluate immunohistochemistry data, and *P* < 0.05 indicates statistically significance differences.

## Results

**Figure 1:**
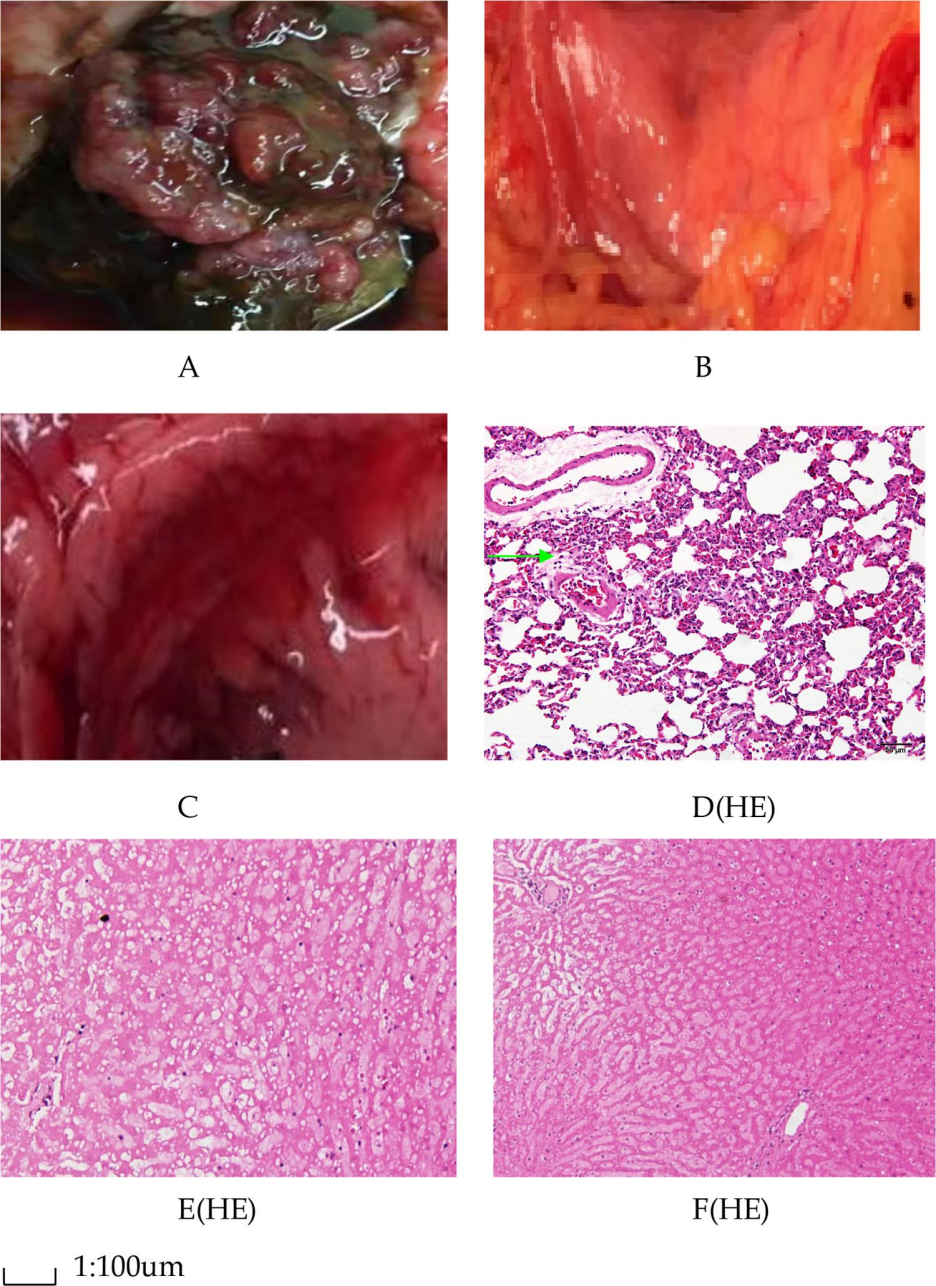
Pathological examination. (**A**) hilar cholangiocarcinoma in experimental group. (**B**) hilar bile duct in control group. (**C**) hilar bile duct in sham operation group. (**D**) tumor cells are indicated by the green arrow in experimental group. HE stain (magnification 100 ×). (**E**) hilar bile duct tissues in control group. HE stain (magnification 100 ×). (**F**) hilar bile duct tissues in sham operation group. HE stain (magnification 100 ×). After 6 weeks, hilar cholangiocarcinomas were detected in hilar bile ducts of 18 rats (90%) in the experimental group, they were cauliflower-like mass in hilar bile ducts. On the contrary only there were mild inflammation and edema in hilar bile ducts in control group and sham operation group. The tumor cells were multinucleated and the mitochondrias were swollen, the cells in control group and sham operation group were mild edema.

From the second week, the rats in the experimental group began to eat less, and their weights decreased compared with control group and sham operation group (Tables 1, 2). One rat in the experimental group died after 6 weeks. There were no fatalities in the control group and sham operation group. After 6 weeks, through pathologic examination we detected hilar cholangiocarcinoma in the hilar bile duct of 18 rats (90%) in the experimental group (Fig. 1). The changes of liver function of the groups are shown in Table 3.

**Table 1:** Daily food-intake (g).

Week	Control group (n = 20)	Sham operation group (n = 20)	Experimental group (n = 20)
2	19.28 ± 0.13	19.32 ± 0.11	19.05 ± 0.08
3	23.71 ± 0.10	24.03 ± 0.12	20.68 ± 0.09
4	27.35 ± 0.08*	28.01 ± 0.15^^^	19.16 ± 0.06
5	28.51 ± 0.12^#^	29.08 ± 0.13^&^	18.63 ± 0.05

*P < 0.05 compared with the experimental group, ^#^*P* < 0.05 compared with the experimental group, ^^^*P* < 0.05 compared with the experimental group, ^&^*P* < 0.05 compared with the experimental group. From the second week, the rats in experimental group began to eat less, and the daily food-intake decreased obviously compared with that in control group and sham operation group.

(SPSS22.0 http://www.downxia.com/downinfo/165523.html).

**Table 2:** Body mass (g).

Week	Control group (n = 20)	Sham operation group (n = 20)	Experimental group (n = 20)
2	209 ± 4.5	208 ± 4.2	207 ± 3.9
3	221 ± 6.1	220 ± 5.3	218 ± 5.7
4	237 ± 6.3*	238 ± 6.2^^^	203 ± 5.5
5	249 ± 7.2^#^	251 ± 6.8^&^	198 ± 4.6

*P < 0.05 compared with the experimental group, ^#^*P* < 0.05 compared with the experimental group, ^^^*P* < 0.05 compared with the experimental group, ^&^*P* < 0.05 compared with the experimental group. From the second week, the rats in experimental group began to eat less, and their body mass decreased obviously compared with that in control group and sham operation group.

(SPSS22.0 http://www.downxia.com/downinfo/165523.html).

**Table 3:** Changes of liver function.

Related indicators	Control group (n = 20)	Sham operation group (n = 20)	Experimental group (n = 20)
(ALT)/(U/L)	82.35 ± 2.71^$^	83.01 ± 2.65^$$^	156.82 ± 5.39
(AST)/(U/L)	89.02 ± 3.06^&^	91.13 ± 2.98^&&^	161.74 ± 5.83
(TC)/(mmol/L)	2.53 ± 0.15*	2.55 ± 0.18**	6.39 ± 0.12
(TBA)/(μmol/L)	1.67 ± 0.06^#^	1.68 ± 0.07^##^	4.83 ± 0.05
(TBIL)/(μmol/L)	3.83 ± 0.05	3.84 ± 0.06	3.82 ± 0.04
(DBIL)/(μmol/L)	0.72 ± 0.03^^^	0.75 ± 0.05^^^^	1.98 ± 0.06

^$^*P* < 0.05 compared with the experimental group, ^&^*P* < 0.05 compared with the experimental group, *P < 0.05 compared with the experimental group, ^#^*P* < 0.05 compared with the experimental group, ^^^*P* < 0.05 compared with the experimental group. ^$$^*P* < 0.05 compared with the experimental group, ^&&^*P* < 0.05 compared with the experimental group, **P < 0.05 compared with the experimental group, ^##^*P* < 0.05 compared with the experimental group, ^^^^*P* < 0.05 compared with the experimental group. The levels of alanine aminotransfease, aspartate transaminase, total cholesterol, total bilirubin, and direct bilirubin in control group and sham operation group were lower compared with those in the experimental group.

ALT: alanine aminotransfease, AST: aspartate transaminase, TC: total cholesterol, TBA: total bile acids, TBIL: Total bilirubin, DBIL: direct bilirubin.

(SPSS22.0 http://www.downxia.com/downinfo/165523.html).

### Analysis of Bsep expression

**Figure 2:**
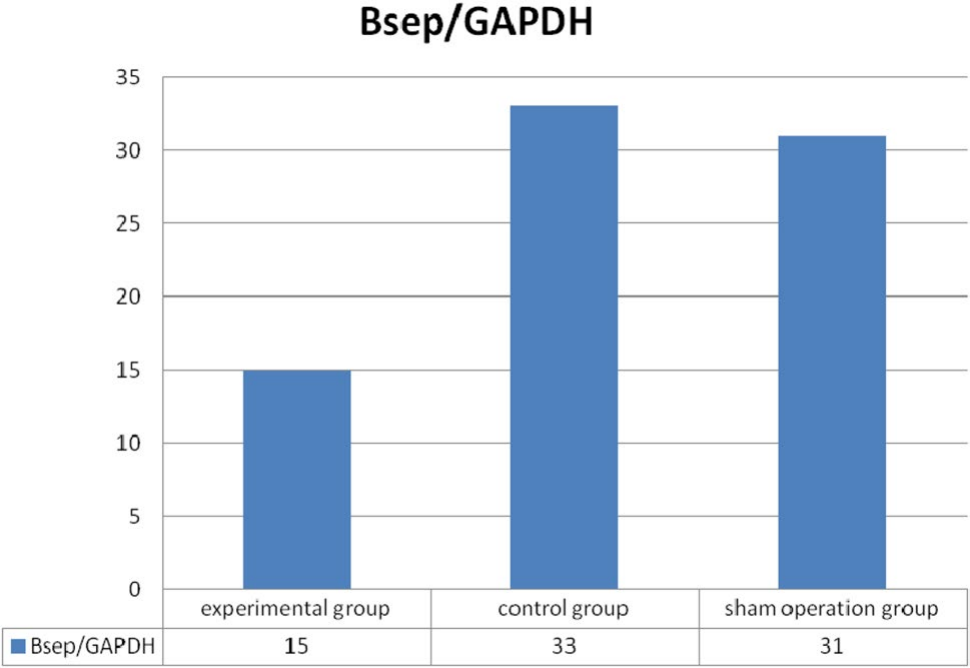
Analysis of *Bsep* mRNA expression. Through RT-PCR we found that in cancerous, control group and sham operation group the *Bsep*/*GAPDH* ratios were 15, 33 and 31, respectively, after eight cycles, and the difference between experimental group and control group was statistically significant (*t* = 2.821, *P* < 0.05), the difference between experimental group and sham operation group was also statistically significant (*t* = 2.793, *P* < 0.05). (SPSS22.0 http://www.downxia.com/downinfo/165523.html).

Through RT-PCR we found that in cancerous, control group and sham operation group the *Bsep*/*Gapdh* ratios were 15, 33 and 31, respectively, after eight cycles, the difference between experimental group and control group was statistically significant (*t* = 2.821, *P* < 0.05), the difference between experimental group and sham operation group was also statistically significant (*t* = 2.793, *P* < 0.05). (Fig. 2).

**Figure 3:**
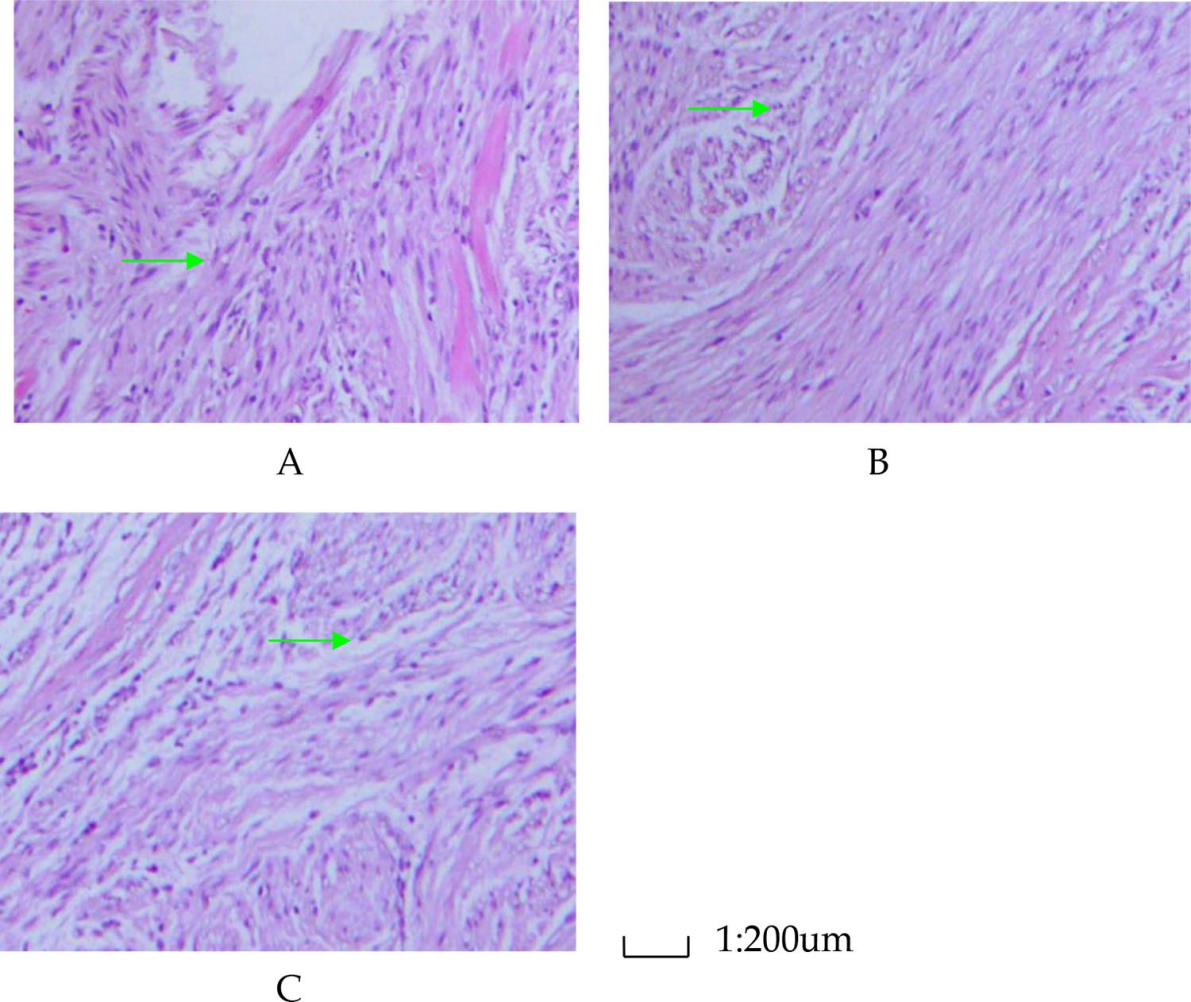
Analysis of *Bsep* expression by immunohistochemical assay. (**A**) *Bsep* expression in hilar cholangiocarcinoma of experimental group (magnification 200 ×). (**B**) *Bsep* expression in normal hilar bile duct from control group (magnification 200 ×). (**C**) *Bsep* expression in normal hilar bile duct from sham operation group (magnification 200 ×). The green arrows indicate *Bsep*. The *Bsep* protein expression which reacted with the anti-*Bsep* antibody were shown by immunohistochemistry. Each section included 4,800 cells. In the experimental group, 1,200 cells (25%) were positive, in the control group, 3,648 cells (76%) were positive and in the sham operation group 3,598 cells (75%) were positive, and this difference was statistically significant. (χ^2^ = 10.28, *P* < 0.05, between experimental group and control group. χ^2^ = 10.26, *P* < 0.05 between experimental group and sham operation group). (SPSS22.0 http://www.downxia.com/downinfo/165523.html).

The *Bsep* protein expression which reacted with the anti-*Bsep* antibody were shown in Fig. 3. Each section included 4800 cells. In the experimental group, 1200 cells (25%) were positive, in the control group, 3648 cells (76%) were positive and in the sham operation group 3598 cells (75%) were positive, and this difference was statistically significant (χ^2^ = 10.28, *P* < 0.05, between experimental group and control group. Χ^2^ = 10.26, *P* < 0.05 between experimental group and sham operation group).

## Discussion

In digestive system the malignant tumors include pancreatic cancer, gastric cancer, primary liver cancer and others. Hilar cholangiocarcinoma exhibit one of the most malignant phenotype. Despite during several years the diagnostic and therapeutic modalities developed in basic and clinical research, the pathogenesis of hilar cholangiocarcinoma, the changes in the pathological and physiological characteristics of tumors, and the changes of the tumor environment are not completely clear^7–10^. Factors that are associated with the pathogenesis of hilar cholangiocarcinoma include Calculus of bile duct, congenital cholangiectasis, Chinese liver fluke and primary sclerosing chola. However, the mechanisms that regulate specific signal transduction pathways associated with the malignant phenotype of hilar cholangiocarcinoma are not clear. For example, hilar cholangiocarcinoma may be caused by specific factors that accumulate and interact each other, therefore which can explain the lack of effective treatments. Usually, if the tumor is small or limited to part of the hilar bile duct, it can be completely resected, patients have a favorable prognosis. However, if the tumor infiltrates surrounding tissues or metastasizes, surgery is impossible. The effects of interventions such as chemotherapy, radiofrequency ablation, radiotherapy, and interventional embolization are limited. Moreover, the rates of recurrence and metastasis are high, and the 5-year survival rate is low. Therefore, more effective diagnostic and therapeutic modalities are urgently required. Although gene therapy is not widely used, it has good prospect. So, we searched for genes involved in the pathogenesis of hilar cholangiocarcinoma that may serve as therapeutic targets^11–13^.

*Bsep* usually expressed on the bile duct, and its function is to transport bile acid. Therefore, *Bsep* plays an important role in bile-acid excretion, bile-acid concentration stabilization, and bile acid into the enterohepatic circulation. We previously found that the FXR expressed in hilar bile duct tissues of rats and that its expression decreased in cholangiocarcinoma tissues. Thus we tried to analyze if the expression levels of *Bsep*, a target gene of FXR, changed similarly in hilar bile duct tissues of rats with hilar cholangiocarcinoma^14–17^. The course of bile acid enterohepatic circulation requires various transporters to interact each other. At first bile acid synthesis by hepatocytes, and bile is exported to the intestinal tract by the bile salt export pump. After bile acid is discharged into the small intestine, approximately 95% of the conjugated bile acid is reabsorbed through the apical sodium-dependent bile acid transporter, ileum bile acid binding protein, and terminal apical sodium-dependent bile acid transporter. Sodium/taurocholate cotransporting polypeptide (Ntcp) mediates approximately 80% bile acids into liver cells, which are again secreted into the bile to formate enterohepatic circulation of bile acids^18–20^.

If the levels of *Bsep* expression are inappropriate, bile secretion disorders may occur, and bile will accumulate in the bile duct. So cholesterol and bile pigments may accumulate in bile ducts, leading to the formation of stones. The long-term presence of a calculus in the bile duct is one of possible causes of promoting the growth of hilar cholangiocarcinoma. Therefore, it is important to gain a better understanding of *Bsep* expression in hilar cholangiocarcinoma tissue^21–24^.

In the present study, we established a rat model of hilar cholangiocarcinoma. After 6 weeks, hilar cholangiocarcinoma developed only in the experimental group, thereby establishing an experimental model for studying QBC939-induced hilar cholangiocarcinoma. The dietary intake and weights of rats in the experimental group were lower than those in control group and sham operation group. The frequency of rats with hilar cholangiocarcinoma was 90%, In the experimental group with hilar cholangiocarcinoma, the levels of alanine aminotransfease, aspartate transaminase, total cholesterol, total bilirubin, and direct bilirubin were higher than those in control group and sham operation group. Simultaneously, muddy stones emerged from the bile ducts of rats in experimental group, and the levels of *Bsep* expression were lower in the rats with hilar cholangiocarcinoma than that in control group and sham operation group. Thus, we speculate that if quantities of bile acids increase in bile ducts, the expression of *Bsep* will increase^25–27^, which will accelerate the secretion of bile acid to maintain its concentrations. However, in hilar cholangiocarcinoma bile acid secretion is greatly reduced, So bile deposits in the bile duct and potential stone formation that induces inflammation of the tissues around the bile duct. Repeated destruction and proliferation of bile duct cells increase the probability of the emergence of cells with malignant phenotype.

There are several problems with the drugs used to treat hilar cholangiocarcinoma. For example, their use is limited because they do not kill all the tumor cells, require large doses, adversely affect the digestive system, and are poorly tolerated. Drugs in the research and development stages do not directly target the genes that contribute to hilar cholangiocarcinoma. The data presented here may enhance our understanding of the molecular basis of hilar cholangiocarcinoma^28–30^. Moreover, our study illuminates that *Bsep* may be a target for new and more effective treatment strategies.
